# Prenatal tobacco smoke exposure and risk for cognitive delays in infants born very premature

**DOI:** 10.1038/s41598-024-51263-9

**Published:** 2024-01-16

**Authors:** E. Melinda Mahabee-Gittens, Nusrat Harun, Meredith Glover, Alonzo T. Folger, Nehal A. Parikh, Cincinnati Infant Neurodevelopment Early Prediction Study (CINEPS) Investigators, Mekibib Altaye, Anita Arnsperger, Traci Beiersdorfer, Kaley Bridgewater, Tanya Cahill, Kim Cecil, Kent Dietrich, Christen Distler, Juanita Dudley, Brianne Georg, Cathy Grisby, Lacey Haas, Lili He, Scott K. Holland, V. S. Priyanka Illapani, Kristin Kirker, Beth M. Kline‐Fath, Hailong Li, Matt Lanier, Stephanie L. Merhar, Greg Muthig, Brenda B. Poindexter, David Russell, Kari Tepe, Leanne Tamm, Julia Thompson, Hui Wang, Jinghua Wang, Brynne Williams, Kelsey Wineland, Sandra Wuertz, Donna Wuest, Weihong Yuan

**Affiliations:** 1 Division of Emergency Medicine, Cincinnati Children’s Hospital Medical Center, Cincinnati, Ohio USA; 2 Department of Pediatrics, College of Medicine, University of Cincinnati, Cincinnati, Ohio USA; 3 Division of Biostatistics and Epidemiology, Cincinnati Children’s Hospital Medical Center, Cincinnati, Ohio USA; 4 Neurodevelopmental Disorders Prevention Center, The Perinatal Institute, Cincinnati Children’s Hospital Medical Center, Cincinnati, Ohio USA; 5 Cincinnati Children’s Hospital Medical Center, Cincinnati, Ohio USA

**Keywords:** Neurology, Risk factors, Neonatology, Preterm birth

## Abstract

Prenatal tobacco smoke exposure (TSE) and prematurity are independent risk factors for abnormal neurodevelopment. The objectives were to compare differences in Bayley-III cognitive, language, and motor scores at 2 years corrected age (CA) in 395 infants born very preterm (≤ 32 weeks gestation) with and without prenatal TSE. We performed multivariable linear regression analyses to examine associations between prenatal TSE and neurodevelopmental outcomes and a mediation analysis to estimate direct effects of prenatal TSE on outcomes and indirect effects through preterm birth. In total, 50 (12.6%) infants had prenatal TSE. Infants with prenatal TSE had lower mean [95% CI] Cognitive score (82.8 [78.6, 87.1]) vs. nonexposed infants (91.7 [90.1, 93.4]). In children with and without prenatal TSE, there were significant differences in mean [95% CI] Language scores (81.7 [76.0, 87.4] vs. 92.4 [90.2, 94.6], respectively) and mean [95% CI] Motor scores (86.5 [82.2, 90.7] vs. 93.4 [91.8, 95.0], respectively); scores remained significant after controlling for confounders. Preterm birth indirectly mediated 9.0% of the total effect of prenatal TSE on Cognitive score (P = NS). However, 91% of the remaining total effect was significant and attributable to TSE’s direct harmful effects on cognitive development (β = − 5.17 [95% CI − 9.97, − 0.38]). The significant association is largely due to TSE’s direct effect on cognitive development and not primarily due to TSE’s indirect effect on preterm birth.

## Introduction

Prenatal tobacco smoke exposure (TSE) is a well-known risk factor for preterm delivery, low birth weight, and adverse neurodevelopmental outcomes in childhood^1–5^. Nevertheless, approximately 5.5% of births in 2020 were to women who smoked during pregnancy, with even higher rates in women who live in rural areas, who have a lower education level, who are unmarried, and who have higher stress levels^3,6^. Since pregnancy is a time when smokers are more likely to quit given their concerns about their unborn child^7,8^, providing information about the long-term effects of maternal tobacco use and prenatal TSE on the unborn child is an important strategy that may further encourage cessation attempts among pregnant women.

There is much research indicating that prenatal TSE in infants who are born at term is associated with adverse neurodevelopmental outcomes such as deficits in cognition, language, and executive function, and increased risk for behavioral problems and mental health conditions^4,5,9–12^. In parallel with this research, it is established that prematurity is also associated with adverse neurodevelopmental outcomes^13–15^. Despite this large body of evidence, there is a gap in our understanding about the neurodevelopmental outcomes in infants who were both born preterm and exposed to prenatal tobacco smoke. Further, infants whose mothers smoke during pregnancy are at increased risk to have concurrent exposures to opioids and other substances^16,17^, to be exposed to in-utero corticosteroids or magnesium, and to have mothers who experienced hypertensive disorders of pregnancy or chorioamnionitis, all of which are also associated with adverse neurodevelopmental consequences^9,18–22^. There are few studies which account for these multiple exposures in preterm infants. Given the concurrent risk factors associated with both prenatal TSE and prematurity and the lack of research on preterm infants who had prenatal TSE, research is needed to examine the indirect mediating effects from direct effects of prenatal TSE on objective measures of neurodevelopment in infants who were born very preterm.

Thus, in this study, we sought to compare differences in neurodevelopmental outcomes conducted at 2 years corrected age in very preterm infants with and without prenatal TSE. The primary study objective was to compare differences in cognitive scores and the secondary objectives were to compare language and motor scores and diagnosis of cerebral palsy in infants who were exposed to prenatal TSE compared to infants with no prenatal TSE. We expected to observe a significant direct adverse effect of maternal smoking during pregnancy on child cognitive development even after we accounted for the indirect mediating effects of premature birth.

## Methods

### Subjects

We recruited a prospective cohort of very preterm infants born at ≤ 32 weeks gestational age (GA) between September 2016 and November 2019 from five level III/IV neonatal intensive care units from the Greater Cincinnati region^23^. To be eligible, infants had no congenital or chromosomal abnormalities that affect the central nervous system. The Strengthening the Reporting of Observational Studies in Epidemiology (STROBE) reporting guidelines for observational studies were followed^24^. All research was performed in accordance with relevant guidelines and regulations.

### Standard protocol approvals, registrations, and patient consents

The institutional review board (IRB) of Cincinnati Children’s Hospital Medical Center approved the study and IRB approval from the other four hospitals was obtained based on an established reliance agreement. Written informed consent was provided by a parent/guardian of each study infant.

### Maternal and infant clinical assessments

#### Social risk scores

Mothers completed assessments at the first study visit at term corrected age which included sociodemographic and prenatal tobacco and substance use history^23^. The following sociodemographic information was obtained to derive an overall social risk score^25^; each of these measures had three potential options that were given a score of 0, 1, or 2, respectively: (1) family income- ≥ $100,000; $40,000–$99,999; or < $40,000; (2) family structure—two caregivers; separated parents with dual custody, or cared for by other intact family; or single caregiver; (3) education of primary caregiver—tertiary education; high school diploma or GED; less than HS diploma or GED; (4) employment status- full-time; part-time, unemployed/receiving a pension; (5) language spoken at home- English only; some English; no English; and (6) maternal age at birth—> age 21; age 18–21; or < age 18. Social risk score was derived by adding these six nominal variables and mothers were categorized as having a high-risk socioeconomic status if their score was ≥ 6. The social risk score is a widely used tool that to assess the association between family social risk and neurodevelopmental outcomes^26–28^.

#### Maternal tobacco and substance use

Mothers reported: (1) tobacco use during pregnancy (Yes/no)—infants of mothers who reported “yes” to tobacco use during pregnancy were considered to have had prenatal TSE; (2) street or prescription drugs used during this pregnancy—infants of mothers who reported “yes” to marijuana, narcotics or narcotic-strength pain medications during pregnancy were considered to have respective prenatal exposures.

#### Pregnancy/delivery and infant information

Research staff collected information on maternal history of: acute histologic chorioamnionitis, using the Redline et al. criteria^29^; hypertensive disorders of pregnancy (HDP)-defined as diagnosis of chronic or gestational hypertension with or without pre-eclampsia; antenatal corticosteroids and magnesium therapy received during the admission prior to this delivery. Infant clinical course, specifically caffeine therapy, postnatal sepsis, bronchopulmonary dysplasia (BPD), postnatal corticosteroids for bronchopulmonary dysplasia, and other variables are described elsewhere in Parikh et al.^23^.

#### Neurodevelopmental outcomes

Children had a repeat visit at 22–26 months corrected age. At that time, Bayley Scales of Infant and Toddler Development (3rd Ed.)^30^ and the Amiel-Tison standardized neurological exam^31^ were administered by certified examiners who completed annual training to ensure reliability to standards developed by the National Institutes of Child Health and Development Neonatal Research Network^32^. Bayley Cognitive, Language, and Motor composite scores are standardized measures with a mean of 100, standard deviation of 15, and a range of 40–160. A few children were untestable due to severe developmental delays; for such children, we assigned a Cognitive score of 54, Language score of 46 and/or a Motor score of 46. Cerebral palsy (CP) was diagnosed using the Amiel-Tison exam as previously described^32^. The primary outcome was the Bayley Cognitive score and the other three measures were secondary outcomes.

### Statistical analysis

We compared baseline maternal and infant characteristics between preterm infants with prenatal TSE compared to those with no prenatal TSE. We performed univariate analyses to evaluate prenatal TSE group differences in our primary outcome and three secondary outcomes. We used linear models for the three continuous outcomes and a logistic regression model for the binary CP outcome. We used multivariable linear regression models for controlling the effects of seven maternal confounders: high-risk socioeconomic status, maternal chorioamnionitis, maternal magnesium therapy, HDP, opioid use during pregnancy, marijuana use during pregnancy, and absent or incomplete course of antenatal steroids.

Preterm birth as measured by gestational age (GA) was significantly different between infants with and without prenatal TSE and preterm birth temporally occurs after TSE. Therefore, GA is likely an indirect mediator between prenatal TSE and neurodevelopmental outcomes. Thus, we performed a causal inference mediation analysis^33^ for outcomes that were significant in multivariable analyses. This allowed us to separate out the direct effect of prenatal TSE on the neurodevelopmental outcomes from the indirect effect attributed to premature birth. This involved two multivariable linear regression models: (1) predict mediator from TSE adjusting for known confounders, (2) predict outcome from TSE adjusting for the mediator and known confounders. We investigated interactions between prenatal TSE and GA on neurodevelopmental outcomes. Since no interaction was detected, we examined the effect of prenatal TSE on neurodevelopmental outcome without considering the interaction term in the outcome model. The indirect (i.e., mediated) effect is the product of the TSE coefficient in the mediator model (1) times the GA coefficient in the outcome regression model (2). The natural direct effect is the coefficient of TSE from the outcome model (2) that includes the mediator^33^. The total effect is the sum of the direct and indirect effects. The percentage of mediated effect was estimated by dividing the TSE indirect effect coefficient by the total effect coefficient and multiplying by 100. The confidence limits were calculated using a normal approximation for the Wald test. Statistical significance was established using P < 0.05. We used SAS (SAS Institute Inc., Cary, NC) version 9.4 to conduct all analyses.

## Results

Of our original cohort of 395 infants, two infants withdrew six months into the study, one died at one year of age, and 341 infants returned and were successfully tested with the Bayley scales (follow-up rate: 86%). In total, 50 (12.7%) of the 395 infants in the cohort had prenatal TSE; 40 (80%) smoked < 10 cigarettes/day. The mean (SD) GA and birthweight was 29.3 (2.5) weeks and 1294.3 (448.9) g, respectively. Further details on maternal and infant history and significant group differences in infants with and without prenatal TSE in the cohort that had a repeat visit at 22–26 months of age (N = 341) are in Table 1.

**Table 1:** Maternal and infant baseline characteristics of very preterm infants with and without prenatal TSE.

Baseline variables n (%)	No prenatal TSE (N = 297)	Prenatal TSE (N = 44)	P
Maternal			
High-risk socioeconomic status	45 (15.2%)	13 (29.5%)	*0.018*
Low income			*0.002*
≥ $100,000	88 (29.6%)	6(13.6%)	
$40,000–$99,999	90 (30.3%)	8 (18.2%)	
< $40,000	119 (40.1%)	30 (68.2%)	
Family structure			*0.002*
Two caregivers	215 (72.4%)	20 (45.5%)	
Separated with dual custody or cared for by other intact family	22 (7.4%)	6 (13.6%)	
One Caregiver	60 (20.2%)	18 (40.9%)	
Education			*0.012*
Tertiary education	222 (74.7%)	24(54.5%)	
High school diploma or GED	61 (20.5%)	15 (34.1%)	
Less than HS or less than GED	14 (4.7%)	5 (11.4%)	
Employment status			*0.0002*
Full-time	169 (56.9%)	13 (29.5%)	
Part-time	46 (15.5%)	5 (11.4%)	
Unemployed/receives a pension	82 (27.6%)	26 (59.1%)	
English spoken at home			0.698
English only	288 (97%)	44 (100%)	
Some English	2 (0.7%)	–	
No English	7 (2.4%)	–	
Maternal age			0.115
> 21 years old	269 (90.6%)	44 (100%)	
18–21 years old	25 (8.4%)	–	
< 18 years old	3 (1%)	–	
Antenatal opioid use	15 (5.1%)	12 (27.3%)	< *0.0001*
Antenatal marijuana use	9 (3%)	9 (20.5%)	< *0.0001*
Antenatal steroids (none or incomplete course)	73 (24.6%)	16 (36.4%)	0.097
Antenatal magnesium	252 (84.8%)	37 (84.1%)	0.896
Histologic chorioamnionitis	83 (27.9%)	14 (31.8%)	0.595
Diabetes (prior to pregnancy or gestational)	53 (17.8%)	7 (15.9%)	0.753
Hypertensive disorders of pregnancy	125 (42.1%)	14 (31.8%)	0.196
Infant			
Male sex	145 (48.8%)	20 (45.5%)	0.677
Multiple births	109 (36.7%)	13 (29.5%)	0.356
Gestational age (weeks), mean (SD)	29.2 (2.5)	28.4 (2.4)	*0.025*
Birth weight Z-score, mean (SD)	0.090 (0.97)	− 0.09 (0.85)	0.163
Small for gestational age	23 (7.7%)	3 (6.8%)	1.000
Apgar score < 5 at 5 min^‡^	35 (11.9%)	7 (16.7%)	0.378
Caffeine therapy	211 (71%)	37 (84.1%)	0.070
Postnatal sepsis	35 (11.8%)	3 (6.8%)	0.445
Bronchopulmonary dysplasia (any severity)	131 (44.1)%	19 (43.2%)	0.908
Postnatal corticosteroids for BPD	33 (11.1%)	6 (13.6%)	0.623
Surgery for NEC or SIP	17 (5.7%)	2 (4.5%)	1.000
Surgery requiring general anesthesia	33 (11.1%)	7 (15.9%)	0.356
Retinopathy of prematurity (any grade)	107 (36%)	21 (47.7%)	0.135

*PMA* postmenstrual age, *NEC* necrotizing enterocolitis, *SIP* spontaneous intestinal perforation.

Significant values are in italics.

^‡^Data unavailable for 3 infants.

There were no statistically significant group differences in variables known to be associated with neurodevelopmental outcomes in prior research: histologic chorioamnionitis, maternal magnesium therapy, absent or incomplete course of antenatal steroids, or HDP^18,20–22,34,35^. Significant group differences were noted between high-risk socioeconomic status, maternal opioid and marijuana use. Multiple known confounders of neurodevelopmental outcomes and statistically significant variables were adjusted for in all multivariable analyses.

Our primary outcome was significantly different between groups in univariate analysis, with infants with prenatal TSE having a mean [95% CI] Bayley Cognitive score of 82.8 [78.6, 87.1] and non-exposed infants scoring 91.7 [90.1, 93.4]. There were also statistically significant differences in the two secondary outcomes of mean [95% CI] Language score in children with and without prenatal TSE (81.7 [76.0, 87.4] vs. 92.4 [90.2, 94.6], respectively) and mean [95% CI] Motor score in children with and without prenatal TSE (86.5 [82.2, 90.7] vs. 93.4 [91.8, 95.0], respectively). In multivariable analyses, Cognitive score remained significant (β [95% CI]: − 5.56 (− 10.55, − 0.57]) after controlling for our seven confounders (Table 2). Statistically significant differences were also observed in Language score between groups (β [95% CI]: − 6.89 (− 13.56, − 0.23]). The estimated means from the adjusted model are: 76.5 and 82.0 (difference is 5.5), exposed vs non-exposed for cognitive scores; 73.5 and 80.4 (difference is 6.9), exposed vs non-exposed for language scores. There were no statistically significant differences in Motor score (P = 0.203) or cerebral palsy diagnosis (P = 0.3395) after adjusting for confounders.

**Table 2:** Relationship between smoking during pregnancy and neurodevelopment outcomes 2 years corrected age in infants born very prematurely in unadjusted analyses and analyses adjusted for confounders.

Neurodevelopmental outcome	Unadjusted		Adjusted*	
	Coefficient (95% CI)	P value	Coefficient (95% CI)	P value
Cognitive score**				
Prenatal TSE	*− 8.89 (− 13.46, − 4.32)*	*0.0002*	− *5.56 (*− *10.55, *− *0.57)*	*0.029*
High-risk socioeconomic status			− *8.55 (*− *12.75, *− *4.35)*	< *0.0001*
Maternal antenatal opioid use			− 4.69 (− 10.65, 1.27)	0.123
Maternal antenatal marijuana use			− *7.58 (*− *14.78, *− *0.39)*	*0.039*
Antenatal steroids (none or incomplete course)			− 2.51 (− 6.21, 1.18)	0.182
Antenatal magnesium therapy			− 1.14 (− 5.84, 3.57)	0.635
Histologic chorioamnionitis			− *3.61 (*− *6.93, *− *0.28)*	*0.034*
Hypertensive disorders of pregnancy			− 2.12 (− 5.32, 1.07)	0.192
Language score**				
Prenatal TSE	− *10.75 (*− *16.85, *− *4.64)*	*0.0006*	− *6.89 (*− *13.56, *− *0.23)*	*0.043*
High-risk socioeconomic status			− *12.69 (*− *18.31, *− *7.07)*	< *0.0001*
Maternal antenatal opioid use			− 2.63 (− 10.60, 5.34)	0.517
Maternal antenatal marijuana use			− 9.38 (− 18.99, 0.24)	0.056
Antenatal steroids (none or incomplete course)			− 2.26 (− 7.22, 2.69)	0.369
Antenatal magnesium therapy			0.20 (− 6.18, 6.57)	0.952
Histologic chorioamnionitis			− *7.07 (*− *11.54, *− *2.60)*	*0.002*
Hypertensive disorders of pregnancy			− 1.85 (− 6.15, 2.45)	0.399

Significant values are in italics.

*Adjusted for opioid use during pregnancy, marijuana during pregnancy, absent or incomplete course of antenatal steroids, antenatal magnesium, hypertensive disorders of pregnancy, histologic chorioamnionitis, and high-risk socioeconomic status.

**Assessed with the Bayley Scales of Infant and Toddler Development (3rd Ed.) Cognitive or Language subtests^30^.

A total of 35 mothers reported street drug use during pregnancy, of which only 13 used drugs other than marijuana. In total, 29 mothers reported alcohol use during pregnancy and two mothers reported that they concurrently used both alcohol and tobacco during pregnancy. To account for the potential confounding effect of maternal alcohol use, we conducted a sensitivity analysis to see if excluding the 29 infants from our analysis who were exposed to maternal alcohol use during pregnancy would meaningfully change the effect of TSE on cognitive and language scores. For the Bayley Cognitive score, the beta coefficient only changed by 10%—from 5.56 (P = 0.046) for the original cohort vs. 5.00 (P = 0.064) for the restricted new model. For the Bayley Language score, the beta coefficient only changed by 5%—from 6.89 for the original cohort vs. 7.19 (P = 0.046) for the alcohol negative cohort (see Supplemental Table 1).

Since birth GA was lower in infants with prenatal TSE (P = 0.028), we conducted mediation analyses to determine if preterm birth/GA demonstrated indirect adverse effects on our two outcomes significant in multivariable analyses, Cognitive and Language scores on the Bayley Scales. For our primary outcome, Cognitive score, GA mediated 9.0% of the effect of smoking during pregnancy, though this was not statistically significant (indirect effect coefficient (95% CI) − 0.51 (− 1.51, 0.49)) (Table 3). The remaining 91% of the effect can be attributed as a direct harmful effect of smoking on cognitive development (indirect effect coefficient (95% CI) − 5.17 (− 9.97, − 0.38)). Thus, most of the adverse effects of smoking during pregnancy was a direct effect on the preterm brain and independent of the effect of premature birth. We did not observe a significant indirect effect of preterm birth on language development. (Table 3). This mediation analysis was also conducted excluding the 29 infants who were exposed to maternal alcohol use during pregnancy (See Supplemental Table 2).

**Table 3:** Results of mediation analysis to separate the total effects of prenatal smoking during pregnancy into indirect effects resulting from preterm birth and direct effects on neurodevelopmental outcomes at 2 years corrected age in infants born very prematurely.

Bayley-III outcome measures at age 2*	Total effect coefficient (95% CI) P value	Direct effect coefficient (95% CI) P value	Indirect effect coefficient (95% CI) P value	% mediated
Cognitive composite score	− *5.68 (*− *10.56, *− *0.80)* *P* = *0.022*	− *5.17 (*− *9.97, *− *0.38)* *P* = *0.034*	− 0.51 (− 1.51, 0.49) P = 0.315	9.0%
Language composite score	− *6.88 (*− *13.44, *− *0.32)* *P* = *0.040*	− 6.43 (− 12.95, 0.09) P = 0.053	− 0.45 (− 1.37, 0.47) P = 0.339	6.5%

Significant values are in italics.

*All analyses were adjusted for the following confounders: opioid use during pregnancy, marijuana during pregnancy, absent or incomplete course of antenatal steroids, antenatal magnesium, hypertensive disorders of pregnancy, chorioamnionitis during pregnancy, and high-risk socioeconomic status^25^.

## Discussion

In this study of a regional cohort of 395 infants born very preterm, we validated prior research that reported that prenatal TSE is associated with adverse cognitive, language, and motor pediatric outcomes^9–12,36–38^. In addition, this study bridges an important research gap as the extant literature on prenatal TSE and children’s neurodevelopmental outcomes has largely focused on children born at term. Thus, this study’s findings add to the growing list of known adverse neurodevelopmental outcomes associated with this preventable prenatal exposure in both term^9–12,36–38^ and preterm infants. In this study, we found that at the corrected age of 2 years old, children who were born very preterm and who were exposed to prenatal tobacco smoke scored 5.5 points lower on their mean Cognitive scores and 6.9 points lower on their mean Language scores compared to unexposed children. Similar findings have been reported in children born at term who were exposed to tobacco smoke^9–12,36,37^. Moreover, after adjusting for antenatal confounders known to be associated with adverse neurodevelopmental outcomes^18,20–22,34,35^ such as chorioamnionitis, we found that both the Cognitive score and the Language score remained statistically significantly different between children in each prenatal TSE group. These differences in Cognitive, Language, and Motor scores in children with prenatal TSE are concerning as they were obtained using the Bayley Scales of Infant and Toddler Development (3rd Ed.)^30^, which is the most common standardized test used in neonatal randomized trials to assess children’s development at this age^39^. This validated tool is known to be associated with children’s future development and is an important tool used to identify children with early developmental delays who may benefit from early intervention^39^.

We conducted a mediation analysis to account for the potential indirect effects of preterm birth on Cognitive score, our primary outcome. We found that preterm birth indirectly mediated 9.0% of the total effect of smoking during pregnancy on the Cognitive score, an effect that was not statistically significant (P = 0.315). However, the remaining 91% of the total effect was significant and can be attributed as a direct harmful effect of smoking on cognitive development (P = 0.034). Thus, smoking during pregnancy has a direct and persistent adverse effect on children’s cognitive development 2 years after birth, independent of the effect of TSE on premature birth or associated low birth weight. We did not identify a harmful effect of antenatal TSE on our secondary outcome of Bayley Motor scores and CP diagnosis. However, our study was likely insufficiently powered to examine this lower prevalence outcome. The potential mechanisms by which nicotine exerts adverse neurodevelopmental has been studied in laboratory research. Nicotine can cross the placenta and binds to neuronal nicotinic acetylcholine receptors (nAChRs), which are found throughout the fetal nervous system and which regulate fetal brain maturation^40,41^. In animal models, nicotine exposure has adverse neurodevelopmental effects resulting in cell damage, decreased cell numbers, impaired synaptic activity, the initiation of apoptosis, and other effects^40–42^.

This study’s findings could potentially be leveraged in tobacco cessation interventions conducted during pregnancy. It is well-known that pregnancy is a time when many female smokers successfully quit smoking or decrease the amount they smoke^7,8^. Several studies indicate that concern about the effects of smoking on their baby’s health and the desire to quit smoking to protect their unborn child may serve as facilitators to help pregnant women quit smoking^43,44^. However, some pregnant mothers report being unclear about whether smoking is truly harmful to their babies^45,46^. Although there is little research related to prenatal TSE and long-term neurodevelopmental outcomes in children who were born preterm, one study of preterm infants born at < 32 weeks GA found poor motor and cognitive outcomes at 2 years corrected age in those who were exposed to prenatal TSE compared to those with no prenatal TSE^47^. However, this study did not conduct a mediation analysis to disentangle the direct effect of TSE on neurodevelopment from its indirect effect on preterm birth/low birth weight. Further, prior research indicates that term infants who were exposed to in-utero TSE and who were small for gestational age born have lower reading and math scores at age 5 compared to unexposed children^48^. Other studies on term infants reported lower Bayley scores in areas such as Motor, Cognition, Language, and Adaptive Behavior at age 2^11,12,37^. Such information from this current study and past studies about the long-term neurodevelopmental effects of maternal tobacco use and prenatal TSE on the unborn child may be an important strategy that may further encourage cessation attempts among pregnant women.

Despite the many strengths of this study which include the examination of a regional cohort of infants born very preterm and the longitudinal nature of our outcomes, there are limitations that should be acknowledged. Prenatal TSE and substance use was assessed via maternal self-reports. However, if mothers underreported TSE, findings would have been even stronger if biochemical validation of TSE and substance use had been obtained. Additionally, when we conducted a sensitivity analysis in which we excluded the 29 infants who were exposed to alcohol during pregnancy, we observed a small but blunted effect on Bayley Language and Cognitive scores which were no longer statistically significant. Thus, future studies are needed to investigate these associations in larger cohorts of preterm infants who had in-utero exposure to alcohol, TSE, or both. Further, although we controlled for the potential effects of important maternal confounders such as antenatal opioid and marijuana use, it is notable that there were statistically significant differences in antenatal opioid and marijuana use in infants in the TSE group. Including these two confounders in our model may be insufficient and residual confounding could still have affected the observed relationships between TSE and neurodevelopmental outcomes; thus, the results should be evaluated with caution. Further, the characteristics of the infants and mothers in this cohort may be dissimilar to other infant cohorts in terms of sociodemographics, tobacco and substance use history and other factors, so it is unknown if our findings are generalizable. Finally, there is some controversy on whether Bayley scales are strongly predictive of future developmental delays in very preterm infants as some research reports poor associations^49,50^ and other reports strong associations, especially when corrected age is used^51,52^. We are now following this cohort up to school age, which will facilitate more robust measures of cognitive and executive functions.

## Conclusions

This study provides further evidence that prenatal TSE is associated with adverse neurodevelopmental outcomes in a longitudinal sample of children born very preterm. Moreover, mediation analyses revealed a large direct effect of prenatal TSE on cognitive scores, which may be associated with future developmental delays. This effect was largely independent of the effect of preterm birth and growth restriction on neurodevelopment. These findings add to the evidence base of the effects of TSE, a preventable exposure, on children’s neurodevelopment. Future work should consider incorporating information about the effects of prenatal TSE on children’s short- and long-term neurodevelopment outcomes into prenatal cessation interventions. Such information may provide further impetus to encourage smokers to quit smoking during pregnancy as a way to improve their unborn child’s future neurodevelopmental outcomes.

### Supplementary Information

Supplementary Tables.

## Data Availability

The datasets used and/or analysed during the current study available from Dr. Nehal Parikh on reasonable request.

## References

[1] Soneji S, Beltran-Sanchez H (2019). Association of maternal cigarette smoking and smoking cessation with preterm birth. JAMA Netw. Open..

[2] Hawsawi AM, Bryant LO, Goodfellow LT (2015). Association between exposure to secondhand smoke during pregnancy and low birthweight: A narrative review. Respir. Care..

[3] Xie S, Monteiro K, Gjelsvik A (2023). The association between adverse birth outcomes and smoking cessation during pregnancy across the United States-43 States and New York City, 2012–2017. Arch. Gynecol. Obstet..

[4] Jamshed L, Perono GA, Jamshed S, Holloway AC (2020). Early life exposure to nicotine: Postnatal metabolic, neurobehavioral and respiratory outcomes and the development of childhood cancers. Toxicol. Sci..

[5] McGrath-Morrow SA (2020). The effects of nicotine on development. Pediatrics..

[6] QuickStats: Percentage of births to mothers who reported smoking cigarettes at any time during pregnancy, by urbanization level* of county of residence—United States, 2020. *MMWR Morb. Mortal. Wkly. Rep*. **70**, 1652. 10.15585/mmwr.mm7047a5 (2021). 10.15585/mmwr.mm7047a5 PMC8612511 34818315

[7] Diamanti A (2019). Smoking cessation in pregnancy: An update for maternity care practitioners. Tob. Induc. Dis..

[8] Gould GS, Havard A, Lim LL, Kumar R, The Psanz Smoking In Pregnancy Expert Group (2020). Exposure to tobacco, environmental tobacco smoke and nicotine in pregnancy: A pragmatic overview of reviews of maternal and child outcomes, effectiveness of interventions and barriers and facilitators to quitting. Int. J. Environ. Res. Public Health..

[9] Lee M (2019). Exposure to prenatal secondhand smoke and early neurodevelopment: Mothers and Children's Environmental Health (MOCEH) study. Environ. Health..

[10] Lee BE (2011). Secondhand smoke exposure during pregnancy and infantile neurodevelopment. Environ. Res..

[11] Christensen GM (2022). In-utero exposure to indoor air pollution or tobacco smoke and cognitive development in a South African birth cohort study. Sci. Total Environ..

[12] He Y, Luo R, Wang T, Gao J, Liu C (2018). Prenatal exposure to environmental tobacco smoke and early development of children in rural Guizhou Province, China. Int. J. Environ. Res. Public Health..

[13] Cha JH (2022). Impact of preterm birth on neurodevelopmental disorders in South Korea: A nationwide population-based study. J. Clin. Med..

[14] Jarjour IT (2015). Neurodevelopmental outcome after extreme prematurity: A review of the literature. Pediatr. Neurol..

[15] Sarda SP, Sarri G, Siffel C (2021). Global prevalence of long-term neurodevelopmental impairment following extremely preterm birth: A systematic literature review. J. Int. Med. Res..

[16] Baer RJ (2019). Risk of preterm and early term birth by maternal drug use. J. Perinatol..

[17] Smith BL (2022). Rates of substance and polysubstance use through universal maternal testing at the time of delivery. J. Perinatol..

[18] Ninan K, Liyanage SK, Murphy KE, Asztalos EV, McDonald SD (2022). Evaluation of long-term outcomes associated with preterm exposure to antenatal corticosteroids: A systematic review and meta-analysis. JAMA Pediatr..

[19] Nygaard E, Slinning K, Moe V, Walhovd KB (2017). Cognitive function of youths born to mothers with opioid and poly-substance abuse problems during pregnancy. Child Neuropsychol..

[20] Jain VG (2022). Acute histologic chorioamnionitis independently and directly increases the risk for brain abnormalities seen on magnetic resonance imaging in very preterm infants. Am. J. Obstet. Gynecol..

[21] Wolf HT (2020). Magnesium sulphate for fetal neuroprotection at imminent risk for preterm delivery: A systematic review with meta-analysis and trial sequential analysis. BJOG..

[22] van Wassenaer AG (2011). Outcome at 4.5 years of children born after expectant management of early-onset hypertensive disorders of pregnancy. Am. J. Obstet. Gynecol..

[23] Parikh NA (2021). Perinatal risk and protective factors in the development of diffuse white matter abnormality on term-equivalent age magnetic resonance imaging in infants born very preterm. J. Pediatr..

[24] von Elm E (2007). The Strengthening the Reporting of Observational Studies in Epidemiology (STROBE) statement: Guidelines for reporting observational studies. Epidemiology..

[25] Roberts G (2008). Rates of early intervention services in very preterm children with developmental disabilities at age 2 years. J. Paediatr. Child Health..

[26] Spittle AJ, Treyvaud K, Lee KJ, Anderson PJ, Doyle LW (2018). The role of social risk in an early preventative care programme for infants born very preterm: A randomized controlled trial. Dev. Med. Child Neurol..

[27] Sapiets SJ, Hastings RP, Totsika V (2023). Predictors of access to early support in families of children with suspected or diagnosed developmental disabilities in the United Kingdom. J. Autism Dev. Disord..

[28] Huf IU (2023). Neurological examination at 32-weeks postmenstrual age predicts 12-month cognitive outcomes in very preterm-born infants. Pediatr. Res..

[29] Redline RW (2003). Amniotic infection syndrome: Nosology and reproducibility of placental reaction patterns. Pediatr. Dev. Pathol..

[30] Bayley N (2006). Bayley Scales of Infant and Toddler Development: Bailey III.

[31] Amiel-Tison C, Gosselin J (2001). Neurological Development from Birth to Six Years: Guide for Examination and Evaluation.

[32] Newman JE (2012). Improving the Neonatal Research Network annual certification for neurologic examination of the 18–22 month child. J. Pediatr..

[33] VanderWeele TJ (2016). Mediation analysis: A practitioner's guide. Annu. Rev. Public Health..

[34] Shi Z (2017). Chorioamnionitis in the development of cerebral palsy: A meta-analysis and systematic review. Pediatrics..

[35] Xing L (2019). Is chorioamnionitis associated with neurodevelopmental outcomes in preterm infants? A systematic review and meta-analysis following PRISMA. Medicine (Baltimore)..

[36] Tsai MS (2017). Children's environmental health based on birth cohort studies of Asia. Sci. Total Environ..

[37] Polanska K (2017). Environmental tobacco smoke exposure during pregnancy and child neurodevelopment. Int. J. Environ. Res. Public Health..

[38] Moore BF (2020). Prenatal exposure to tobacco and offspring neurocognitive development in the Healthy Start study. J. Pediatr..

[39] Del Rosario C, Slevin M, Molloy EJ, Quigley J, Nixon E (2021). How to use the Bayley Scales of Infant and Toddler Development. Arch. Dis. Child. Educ. Pract. Ed..

[40] Dwyer JB, Broide RS, Leslie FM (2008). Nicotine and brain development. Birth Defects Res. C Embryo Today..

[41] Dwyer JB, McQuown SC, Leslie FM (2009). The dynamic effects of nicotine on the developing brain. Pharmacol. Ther..

[42] England LJ, Bunnell RE, Pechacek TF, Tong VT, McAfee TA (2015). Nicotine and the developing human: A neglected element in the electronic cigarette debate. Am. J. Prev. Med..

[43] Flemming K, McCaughan D, Angus K, Graham H (2015). Qualitative systematic review: Barriers and facilitators to smoking cessation experienced by women in pregnancy and following childbirth. J. Adv. Nurs..

[44] Bauld L (2017). Barriers to and facilitators of smoking cessation in pregnancy and following childbirth: Literature review and qualitative study. Health Technol. Assess..

[45] Fletcher C (2022). Isolation, marginalisation and disempowerment—Understanding how interactions with health providers can influence smoking cessation in pregnancy. BMC Pregnancy Childbirth..

[46] Goszczynska E, Knol-Michalowska K, Petrykowska A (2016). How do pregnant women justify smoking? A qualitative study with implications for nurses' and midwives' anti-tobacco interventions. J. Adv. Nurs..

[47] Kiechl-Kohlendorfer U (2010). Smoking in pregnancy: A risk factor for adverse neurodevelopmental outcome in preterm infants?. Acta Paediatr..

[48] Li X (2016). Etiological subgroups of small-for-gestational-age: Differential neurodevelopmental outcomes. PLOS One..

[49] Spencer-Smith MM, Spittle AJ, Lee KJ, Doyle LW, Anderson PJ (2015). Bayley-III Cognitive and Language Scales in preterm children. Pediatrics..

[50] Anderson PJ, Burnett A (2017). Assessing developmental delay in early childhood—Concerns with the Bayley-III scales. Clin. Neuropsychol..

[51] Mansson J (2021). The ability of Bayley-III scores to predict later intelligence in children born extremely preterm. Acta Paediatr..

[52] Morsan V, Fantoni C, Tallandini MA (2018). Age correction in cognitive, linguistic, and motor domains for infants born preterm: An analysis of the Bayley Scales of Infant and Toddler Development, developmental patterns. Dev. Med. Child Neurol..

