# Supplementary material for: Prenatal tobacco smoke exposure and risk for cognitive delays in infants born very premature
Source: Sci Rep. 2024 Jan 16;14:1397. doi: 10.1038/s41598-024-51263-9 (PMC10791619; doi:10.1038/s41598-024-51263-9)
Supplement: Supplementary file 1 — Supplementary Tables. [file 41598_2024_51263_MOESM1_ESM.docx]

**Supplemental Table 1**: Sensitivity analysis on the effect of TSE and Bayley Cognitive and Language Scores excluding 29 children who had antenatal alcohol exposure.

|  | **Unadjusted** |  | **Adjusted*** |  |
| --- | --- | --- | --- | --- |
| **Neurodevelopmental Outcome** | Coefficient  (95% CI) | P value | Coefficient  (95% CI) | P value |
| **Cognitive score**** |  |  |  |  |
| Prenatal TSE | *-7.88*  *(-12.75, -3.00)* | *<0.001* | -5.00  (-10.31, 0.31) | 0.065 |
| High-risk socioeconomic status |  |  | *-9.01*  *-(13.50, -4.52)* | *<.001* |
| Maternal antenatal opioid use |  |  | -4.12  (-10.74, 2.50) | 0.221 |
| Maternal antenatal marijuana use |  |  | -7.96  (-16.35, 0.42) | 0.063 |
| Antenatal steroids (none or incomplete course) |  |  | -2.66  (-6.62, 1.30) | 0.187 |
| Antenatal magnesium therapy |  |  | -1.61  (-6.44, 3.23) | 0.514 |
| Histologic chorioamnionitis |  |  | -3.49  (-7.05, 0.07) | 0.054 |
| Hypertensive disorders of pregnancy |  |  | -1.4592  (-4.86, 1.94) | 0.400 |
| **Language score**** |  |  |  |  |
| Prenatal TSE | *-10.24*  *(-16.71, -3,77)* | *0.002* | *-7.19*  *(-14.26, -0.12)* | *0.046* |
| High-risk socioeconomic status |  |  | *-12.97*  *(-18.94, -6.99)* | *<.001* |
| Maternal antenatal opioid use |  |  | -1.63  (-10.44, 7.18) | 0.716 |
| Maternal antenatal marijuana use |  |  | -9.79  (-20.96, 1.37) | 0.085 |
| Antenatal steroids (none or incomplete course) |  |  | -2.86  -(8.15, 2.42) | 0.287 |
| Antenatal magnesium therapy |  |  | -0.37  (-6.90, 6.16) | 0.912 |
| Histologic chorioamnionitis |  |  | *-5.96*  *(-10.73, -1.19)* | *0.014* |
| Hypertensive disorders of pregnancy |  |  | -1.45  (-6.01, 3.10) | 0.530 |

*Adjusted for opioid use during pregnancy, marijuana during pregnancy, absent or incomplete course of antenatal steroids, antenatal magnesium, hypertensive disorders of pregnancy, histologic chorioamnionitis, and high-risk socioeconomic status.

**Assessed with the Bayley Scales of Infant and Toddler Development (3^rd^ Ed.) Cognitive or Language subtests.[[30](#_ENREF_30)]

**Supplemental Table 2.** Results of mediation analysis to separate the total effects of prenatal smoking during pregnancy into indirect effects resulting from preterm birth and direct effects on neurodevelopmental outcomes at 2 years corrected age in infants born very prematurely, excluding 29 children who had antenatal alcohol exposure.

| **Bayley-III outcome measures at age 2*** | **Total Effect Coefficient**  **(95%CI)**  **P value** | **Direct Effect Coefficient**  **(95% CI)**  **P value** | **Indirect Effect Coefficient**  **(95% CI)**  **P value** | **% Mediated** |
| --- | --- | --- | --- | --- |
| Cognitive composite score | -5.12  (-10.30, 0.06)  P=0.053 | -4.80  (-9.89, 0.29)  P=0.064 | -0.32  (-1.33, 0.70)  P=0.541 | 6.2% |
| Language composite score | *-7.19*  *(-14.13, -0.25)*  *P=0.042* | -6.89  (-13.77, -0.01)  P=0.049 | -0.30  (-1.26, 0.67)  P=0.545 | 4.1% |

*All analyses were adjusted for the following confounders: opioid use during pregnancy, marijuana during pregnancy, absent or incomplete course of antenatal steroids, antenatal magnesium, hypertensive disorders of pregnancy, chorioamnionitis during pregnancy, and high-risk socioeconomic status.[[25](#_ENREF_25)]
